# Effect of Intraoperative Active Warming Initiated at Anesthesia Induction on Core Temperature, Postoperative Pain and Agitation in Laparoscopic Cholecystectomy: A Randomized Controlled Trial

**DOI:** 10.3390/medicina62010175

**Published:** 2026-01-15

**Authors:** Andaç Dedeoğlu, Fatma Acil, Okan Andıç, Mehmet Özkılıç

**Affiliations:** Department of Anesthesiology and Reanimation, TR HSU Diyarbakır Gazi Yaşargil TRH, 21070 Diyarbakır, Turkey; acilfatma@gmail.com (F.A.); okan_andic@hotmail.com (O.A.); ozkilicmemet@gmail.com (M.Ö.)

**Keywords:** active warming, peri-induction period, laparoscopic cholecystectomy, postoperative agitation, postoperative pain, perioperative hypothermia

## Abstract

*Background and Objectives*: Inadvertent perioperative hypothermia is a common and clinically significant complication during laparoscopic surgery, leading to pain, agitation, shivering, and delayed recovery. This randomized controlled trial evaluated the effect of peri-induction active warming with an electric resistive blanket on postoperative pain and agitation—the primary outcomes—compared with passive insulation. *Materials and Methods*: This study was registered at ClinicalTrials.gov (Identifier: NCT06022926; date of registration: 15 August 2023) prior to the enrollment of the first patient. One hundred and thirty-two American Society of Anesthesiologists I–II adults undergoing laparoscopic cholecystectomy were randomly allocated (1:1) to two groups: one received active warming with a resistive carbon fiber underbody blanket (Group 1), and the other received passive insulation (Group 2). The tympanic core temperature was measured at four perioperative time points (TT1–TT4). Postoperative agitation (Riker Sedation–Agitation Scale, RSAS) and pain (Numerical Rating Scale, NRS) were assessed 20 min after extubation in the post-anesthesia care unit (PACU). Secondary outcomes included intraoperative and postoperative temperature, postoperative shivering, adverse events (bradycardia, tachycardia, hypotension, hypertension, postoperative nausea and vomiting, and respiratory depression), and the PACU length of stay. *Results:* Baseline core temperatures (TT1) were similar between the groups (36.5 ± 0.55 °C vs. 36.6 ± 0.54 °C; *p* = 1.00). However, mean core temperatures at TT2, TT3, and TT4 were significantly higher in the active warming group compared with the control group (TT2: 36.7 ± 0.53 °C vs. 36.5 ± 0.54 °C; TT3: 36.6 ± 0.49 °C vs. 36.4 ± 0.54 °C; TT4: 36.6 ± 0.51 °C vs. 36.2 ± 0.52 °C; all *p* < 0.001). Active warming markedly reduced postoperative agitation (RSAS ≥ 5: 3.1% vs. 19.4%, *p* = 0.004) and pain (NRS ≥ 4: 15.4% vs. 49.3%, *p* < 0.001). The incidence of shivering was lower (20.0% vs. 46.3%, *p* = 0.006), and the PACU stay was shorter (24 [23–28] min vs. 35 [30–40] min, *p* < 0.001) with active warming. No significant differences in adverse events were observed between groups. Logistic regression identified the intraoperative fentanyl dose as a predictor of agitation and identified shivering and the PACU duration as predictors of pain. *Conclusions:* Peri-induction active warming effectively maintained normothermia and improved recovery quality by reducing postoperative agitation, pain, shivering, and PACU stays without increasing adverse events. It should be considered a standard component of thermal management in short- and medium-duration laparoscopic surgeries.

## 1. Introduction

Laparoscopic cholecystectomy is a commonly performed surgical procedure due to its minimally invasive nature, which provides advantages such as less postoperative pain and faster recoveries. However, the use of cold and dry carbon dioxide (CO_2_) during laparoscopy, combined with the thermoregulatory threshold-lowering effects of anesthesia, increases the risk of intraoperative hypothermia [1,2]. This condition is further exacerbated by additional factors such as the operating room environment, irrigation fluids, and drug-induced core-to-peripheral heat redistribution [3,4].

Perioperative hypothermia, defined as a core body temperature below 36.0 °C, can occur in 25–70% of surgical procedures [1]. During the first hour of the intraoperative period, anesthesia-induced core-to-peripheral heat redistribution is the primary cause of temperature decline, with most heat loss occurring in this phase [5]. Hypothermia may lead to serious complications, including cardiac morbidity, increased bleeding and coagulopathy, delayed wound healing, surgical site infection, altered drug metabolism, and delayed emergence from anesthesia [2,3,6]. In addition, it may increase shivering, pain, and agitation, thereby reducing patient comfort and recovery quality [1,7,8].

Active (forced-air warming, electric blankets, resistive/conductive systems, and fluid warmers) and passive insulation methods are used to prevent hypothermia [5,9,10]. It has been reported that preoperative and intraoperative warming strategies can reduce redistribution-related heat loss and even accelerate recovery by decreasing analgesic requirements [3,5,8,9]. However, the literature remains heterogeneous regarding the optimal warming methods and combinations in short- and medium-duration laparoscopic procedures [6,10,11]. Moreover, the benefit of using heated and humidified CO_2_ remains controversial [6,12]. Despite the widespread use of forced-air warming, there remains limited evidence regarding whether a brief peri-induction resistive warming approach can provide meaningful clinical recovery benefits in short-duration laparoscopic surgery, such as laparoscopic cholecystectomy.

The number of randomized controlled studies evaluating the efficacy of extrinsic warming devices (e.g., electric blankets) in reducing postoperative agitation and pain is limited [1,7,9,10]. Unintended perioperative hypothermia negatively affects patient comfort by causing postoperative shivering and pain, whereas temperature maintenance strategies not only help to sustain normothermia but may also enhance comfort by reducing analgesic requirements. It is thought that controlling hypothermia may decrease postoperative pain and agitation; however, this relationship has not yet been clearly established [7,8].

In the present study, we hypothesized that peri-induction active warming with an electric blanket would more effectively maintain intraoperative and postoperative normothermia and reduce postoperative pain and agitation compared with passive insulation during laparoscopic cholecystectomy. The primary outcomes of work were postoperative agitation and pain, while the secondary outcomes included intraoperative and postoperative tympanic temperature, postoperative shivering, adverse events (such as bradycardia, tachycardia, hypotension, hypertension, postoperative nausea and vomiting, and respiratory depression), and the length of stay in the post-anesthesia care unit (PACU).

## 2. Materials and Methods

### 2.1. Study Design, Population, and Data

This project followed the Consolidated Standards of Reporting Trials guidelines. This prospective, randomized, controlled study was conducted after obtaining approval from the local ethics committee and was registered at ClinicalTrials.gov (Identifier: NCT06022926; date of registration: 15 August 2023) prior to the enrollment of the first patient. It was carried out between October 2023 and August 2024. All patients were verbally informed about the study and signed a written informed consent form. This study was carefully designed in accordance with the ethical principles outlined in the Declaration of Helsinki (2013), and the original study protocol was strictly followed and can be provided by the authors upon request.

Patients aged 18–65 years who underwent laparoscopic cholecystectomy under general anesthesia at TR HSU Diyarbakır Gazi Yaşargil Training and Research Hospital were included in this study. Eligible participants had an American Society of Anesthesiologists (ASA) physical status score of I–II, a preoperative body temperature between 36 °C and 38 °C, and an expected surgical duration of 30–60 min and were transferred to the PACU after surgery.

Emergency, revision, or converted open surgeries, as well as cases transferred to the intensive care unit or with a surgical duration exceeding one hour, were excluded. Additional exclusion criteria included pregnancy; obesity (BMI > 30–35 kg/m^2^); baseline temperature above 37.8 °C; endocrine, peripheral vascular, neurological, or neuropsychiatric disorders; active infection; blood loss > 50 mL or need for transfusion; use of vasoactive drugs; withdrawal of consent; and development of complications that interfered with data collection.

Patients were randomly assigned in a 1:1 ratio to the active warming or control group using a computer-generated randomization sequence created by an independent investigator who was not involved in patient recruitment, intraoperative management, or outcome assessment. Allocation concealment was ensured using sequentially numbered, opaque, sealed, and tamper-proof envelopes prepared before the start of the study. Each envelope was opened in the operating room only after patient enrollment, immediately prior to anesthesia induction, by an anesthesiologist responsible for implementing the warming intervention and not involved in postoperative outcome evaluation.

Blinding was ensured at multiple levels. Although blinding of the anesthesiologists performing intraoperative warming was not feasible, patients, surgeons, anesthesiologists assessing postoperative outcomes, follow-up investigators, and data analysts were all blinded to group allocation.

In Group 1, patients received active warming with a resistive carbon fiber underbody blanket, starting at anesthesia induction and continuing until the end of surgery. Group 2 served as the control group and received conventional passive insulation only.

### 2.2. Intraoperative Anesthesia Management

On the day of surgery, after an 8-h preoperative fasting period, patients were brought to the operating room. The ambient temperature of the operating room was maintained at 22–24 °C, with a relative humidity of 40–60%. Monitoring was performed according to the ASA standards and included electrocardiography, peripheral oxygen saturation, and noninvasive blood pressure measurements. Two intravenous lines were established using 20-gauge cannulas inserted into the antecubital veins. Throughout the intraoperative and PACU periods, intravenous crystalloid fluids stored at room temperature (22–24 °C) were administered at the same standardized infusion rate of 3 mL/kg/h in both groups, and no fluid warming devices were used.

For anesthesia induction, patients received 0.1 mg/kg of midazolam, 2–3 mg/kg of propofol, 2 µg/kg of fentanyl, and 0.6 mg/kg of rocuronium intravenously. After achieving adequate muscle relaxation, endotracheal intubation was performed using appropriately sized tubes. General anesthesia was maintained with sevoflurane at a concentration of 1 MAC in a mixture of 50% oxygen and air, delivered at a flow rate of 3 L/min. If mean arterial pressure increased by ≥20% during surgery, a rescue dose of 50 µg fentanyl was administered intravenously.

All laparoscopic cholecystectomy procedures in this study were performed using a standardized surgical technique, with a consistent number and size of trocar incisions across patients, in accordance with routine institutional practice. Therefore, incision dimensions did not vary between groups and were not expected to confound postoperative pain outcomes. In all cases, pneumoperitoneum was maintained at a standard intra-abdominal pressure routinely used for laparoscopic cholecystectomy, without intentional variation between patients or study groups. As intra-abdominal pressure was applied uniformly, it was not analyzed as a separate variable and was unlikely to influence between-group differences in postoperative pain.

Thirty minutes before the end of surgery, all patients received 100 mg of intravenous tramadol for analgesia and 0.1 mg/kg of intravenous ondansetron for prevention of postoperative nausea and vomiting (PONV). At the completion of surgery, neuromuscular blockade was reversed with 0.01 mg/kg of intravenous atropine and 0.02 mg/kg of intravenous neostigmine methylsulfate. Patients were then extubated and transferred to the PACU upon awakening from anesthesia. In the PACU, 100 mg of intravenous tramadol was administered if the patient’s Numerical Rating Scale (NRS) pain score was ≥5.

### 2.3. Perioperative Warming Management

In both groups, patients were covered with a single cotton blanket throughout the perioperative period (preparation, intraoperative, transfer, and PACU phases) to provide passive insulation. In addition, a resistive carbon fiber underbody warming blanket (Medwarm 300, İstanbul Medikal, İstanbul, Türkiye) set to 38 °C was placed beneath a cotton sheet on the operating table for all patients.

In Group 1, the Medwarm device was turned on at the start of anesthesia induction (≤1 min from induction), and active warming continued until the patient was extubated. Tympanic body temperatures exceeding 37.8 °C were considered indicative of hyperthermia [13]. The warming system was turned off when the tympanic temperature reached 37.8 °C. In Group 2, the Medwarm device remained switched off.

### 2.4. Outcome Measures and Data Collection

The perioperative demographic characteristics of each patient (age, sex, BMI, and ASA physical status) were recorded. Intraoperative variables, including anesthesia duration, surgical duration, and total intraoperative fentanyl consumption, were also documented. Measurements were obtained at the following time points: immediately before anesthesia induction (T1); 20 min after induction (T2); after extubation (T3); and upon discharge from the PACU (T4).

Tympanic temperature was measured at T1, T2, T3, and T4 (TT1, TT2, TT3, and TT4). Esophageal or nasopharyngeal temperature probes provide highly accurate measurements of core temperature; however, their routine use may not be feasible in short-duration laparoscopic procedures involving low-risk (ASA I–II) patients. Therefore, bilaterally averaged tympanic temperature measurement was used as a validated and noninvasive surrogate for core temperature in the perioperative and PACU settings. Previous studies have demonstrated that infrared tympanic thermometry correlates well with core temperature and is sufficiently reliable for detecting clinically relevant perioperative hypothermia and thermal trends, particularly when measurements are performed bilaterally and averaged [2,10]. Nevertheless, tympanic temperature measurement has inherent limitations, including susceptibility to ambient temperature, probe positioning, and inter-observer variability. Accordingly, our findings should be interpreted in light of these constraints. However, because the same standardized measurement protocol and device were consistently applied across both study groups, any potential measurement bias is unlikely to have affected the between-group comparisons.

Postoperative shivering, agitation, and pain were evaluated 20 min after extubation in the PACU (T20PACU).

The primary outcomes were postoperative agitation and pain. Secondary outcomes included tympanic temperature, postoperative shivering, adverse events (such as cardiovascular complications, postoperative nausea and vomiting, and respiratory depression), and length of stay in the PACU (defined as the time required to reach a Modified Aldrete Score ≥ 9). Tympanic temperature was evaluated as a secondary outcome to provide physiological support for the primary outcomes (postoperative shivering, agitation, and pain), since maintaining core temperature is a key determinant of these clinical consequences.

Postoperative analgesic effectiveness was assessed using the NRS, in which pain intensity was rated from 0 to 10, with 0 representing no pain and 10 indicating the most severe pain. The NRS is widely validated, easy to administer, and particularly suitable for early postoperative assessment in the PACU, as it requires less cognitive and motor coordination than the Visual Analog Scale (VAS) and allows rapid verbal reporting in patients recovering from general anesthesia. Shivering was graded using the following 5-point scale: 0 = no shivering; 1 = piloerection or peripheral vasoconstriction but no visible shivering; 2 = muscular activity in only 1 muscle group; 3 = muscular activity in more than 1 muscle group but not generalized; and 4 = shivering involving the whole body [3].

Agitation during the period between extubation and discharge from the PACU was assessed using the Riker Sedation–Agitation Scale (RSAS): 1 = unresponsive—minimal or no response to painful stimuli, unable to communicate or follow commands; 2 = very sedated—arouses to physical stimuli but does not follow commands, unable to communicate, spontaneous movement present; 3 = sedated—difficult to arouse but awakens to verbal stimuli or follows simple commands; 4 = calm and cooperative—calm, easily arousable, follows commands; 5 = agitated—restless, mildly agitated, attempts to sit up; 6 = very agitated—does not calm despite reassurance, bites endotracheal tube; and 7 = dangerously agitated—pulls at tubes or catheters, climbs out of bed, strikes staff [14].

Body temperature was measured at the tympanic membrane using an infrared tympanic thermometer (Thermoscan7, IRT 6520; Braun GmbH, Kronberg, Germany). The right and left tympanic temperatures were measured separately, and the average of the two values was recorded.

### 2.5. Statistical Analysis

The sample size was calculated based on one of the primary clinical outcomes, postoperative pain. An a priori power analysis was performed using G*Power version 3.1.9.4 (Universität Kiel, Kiel, Germany) for a two-tailed independent samples *t*-test with α = 0.05, power = 0.80, and an allocation ratio of 1:1. A moderate effect size (Cohen’s d = 0.5), corresponding to approximately a 1-point difference in early postoperative pain scores reported in a comparable randomized controlled trial evaluating perioperative active warming in children, was assumed [7]. Based on these parameters, the minimum required sample size was 128 patients (64 per group), and 140 patients were planned to allow for potential exclusions or dropouts.

Statistical analyses were performed using Jamovi version 2.5.5 (The Jamovi Project, Sydney, Australia), and graphical visualizations were generated in R version 4.4.0 (R Foundation for Statistical Computing, Vienna, Austria) using the ggplot2 package (version 3.5.1).

Continuous variables were assessed for normality using the Shapiro–Wilk test and are presented as mean ± standard deviation (SD) or median [interquartile range, IQR], as appropriate. Primary outcomes (postoperative pain and agitation) were compared between the two groups using the independent samples *t*-test for normally distributed variables or the Mann–Whitney U test otherwise. Secondary outcomes, including perioperative tympanic temperature, were analyzed using repeated-measures ANOVA with Greenhouse–Geisser correction to account for violations of sphericity. Where significant effects were detected, Bonferroni-adjusted post hoc comparisons were applied. Effect sizes were reported as partial eta squared (ηp^2^). Categorical variables are presented as numbers (percentages) and were analyzed using the chi-square test or Fisher’s exact test, as appropriate.

To determine independent predictors of clinically relevant postoperative agitation (RSAS ≥ 5) and moderate-to-severe pain (NRS ≥ 4), binary logistic regression analyses were conducted. Variables with biological plausibility and those demonstrating significance in univariate analyses (*p* < 0.10) were included in the multivariate models. To control for potential confounding, BMI and anesthesia duration were incorporated as covariates in additional multivariate logistic regression analyses. Results are presented as odds ratios (ORs) with 95% confidence intervals (CIs), and a *p*-value < 0.05 was considered statistically significant.

## 3. Results

### 3.1. Patient Enrollment and Flow

**Figure 1:**
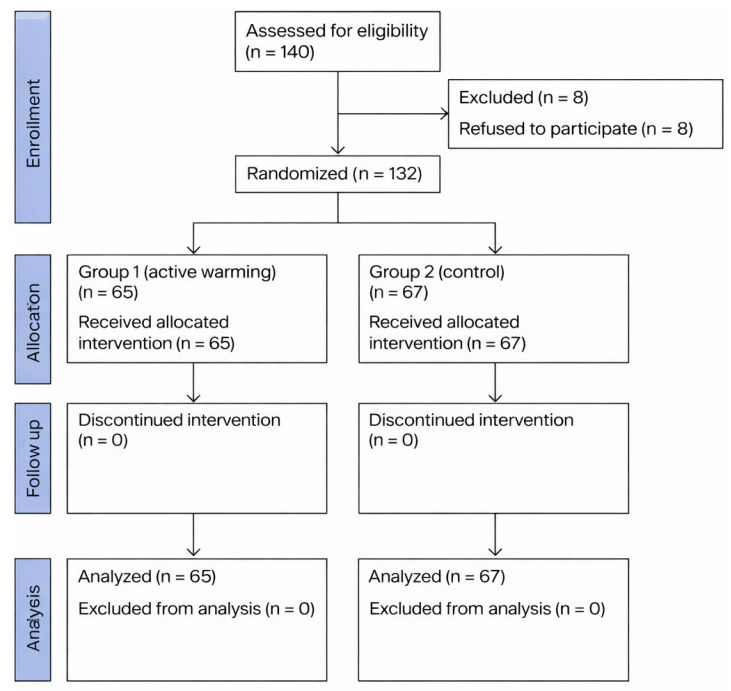
CONSORT flowchart of patient allocation to active warming and control groups.

A CONSORT flow diagram displays the patient enrollment, randomization, allocation, follow-up, and analysis. A total of 140 patients were screened for eligibility, of whom 8 declined to participate. The remaining 132 patients met the inclusion criteria and were randomized into two groups: the active warming group (Group 1, *n* = 65) and the control group (Group 2, *n* = 67). All randomized patients received the allocated intervention and completed the study protocol without protocol deviations or loss to follow-up (Figure 1).

### 3.2. Baseline Characteristics

Baseline demographic and intraoperative characteristics were comparable between the two groups with respect to age, sex distribution, ASA classification, surgical duration, and intraoperative fentanyl consumption. Body mass index was higher in Group 2, whereas anesthesia duration was slightly longer in Group 1 (*p* = 0.013 and *p* = 0.023, respectively). The proportion of patients requiring rescue fentanyl did not differ between groups (12.3% vs. 14.9%, *p* = 0.695) (Table 1).

### 3.3. Perioperative Thermal Profiles

After confirming baseline comparability between groups, subsequent analyses focused on perioperative thermal profiles. Baseline core temperatures (TT1) were similar between the groups. However, mean core temperatures at TT2, TT3, and TT4 were significantly higher in the active warming group compared with the control group, as detailed in Table 2.

**Figure 2:**
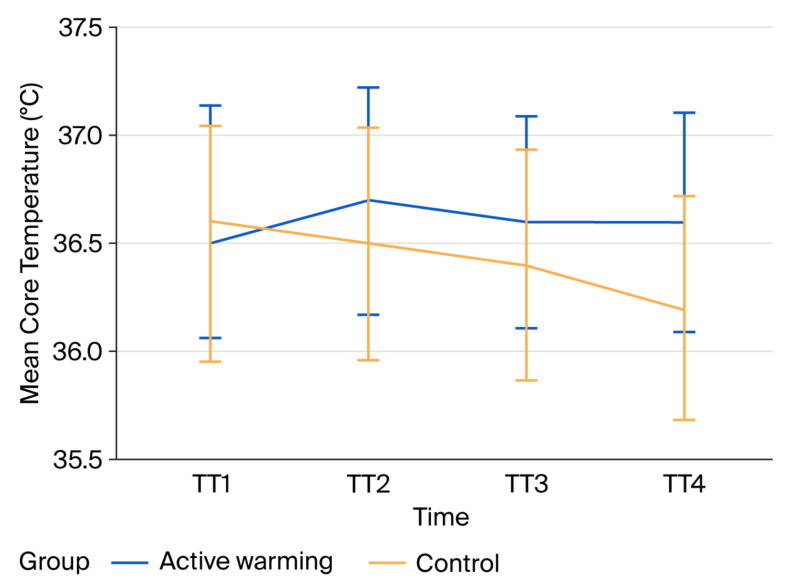
Mean core temperature over time in active warming and control groups.

To assess the effect of active warming on intraoperative temperature trajectories, changes in the mean core temperature over time were analyzed. The analysis identified a significant effect of time (*p* < 0.001); a significant group effect, favoring active warming (*p* = 0.039); and a highly significant time × group interaction (*p* < 0.001), demonstrating greater intraoperative thermal stability in Group 1 (Table 2, Figure 2). The magnitude of this effect was clinically meaningful, with a large time × group interaction effect size (partial η^2^ = 0.440), indicating that active warming substantially improved intraoperative thermal stability rather than producing a marginal statistical difference.

### 3.4. Primary Outcomes

Clinically relevant agitation (RSAS ≥ 5) was more frequent in Group 2 than in Group 1 in the unadjusted analysis (19.4% vs. 3.1%; χ^2^ test, *p* = 0.004). However, this association was no longer statistically significant after multivariable adjustment (adjusted OR 6.12, 95% CI 0.64–58.68; *p* = 0.116). Similarly, the proportion of patients experiencing moderate-to-severe pain (NRS ≥ 4) was significantly higher in Group 2 in the unadjusted analysis (49.3% vs. 15.4%; χ^2^ test, *p* < 0.001), but this difference did not persist after multivariable adjustment (adjusted OR 0.13, 95% CI 0.01–2.48; *p* = 0.176). From a clinical perspective, active warming was associated with a marked reduction in the incidence of both postoperative agitation and moderate-to-severe pain, reflecting a substantial improvement in early recovery quality rather than a solely statistical difference (Table 3).

### 3.5. Secondary Outcomes

The overall incidence of adverse events in the PACU did not differ significantly between groups (*p* = 0.811). Patients receiving active warming experienced a smoother postoperative recovery, characterized by reduced shivering severity and shorter PACU stays compared with controls (Table 4).

### 3.6. Logistic Regression Analysis

To further identify independent factors associated with postoperative outcomes, a multivariate logistic regression analysis was conducted. Logistic regression analysis demonstrated adequate model fit for both outcomes.

For postoperative agitation (RSAS ≥ 5), model fit was satisfactory (Nagelkerke R^2^ = 0.489; Hosmer–Lemeshow χ^2^(8) = 6.72, *p* = 0.567). In the final model, intraoperative fentanyl use was significantly associated with postoperative agitation (OR = 1.05, 95% CI 1.02–1.10; *p* = 0.006), while age, BMI, anesthesia duration, and PACU stay duration were not significant predictors (*p* > 0.05).

Similarly, the model for moderate-to-severe postoperative pain (NRS ≥ 4) was also a satisfactory fit (Nagelkerke R^2^ = 0.871; Hosmer–Lemeshow χ^2^(8) = 4.11, *p* = 0.847). Shivering in the PACU (OR = 0.03, 95% CI 0.01–0.27; *p* = 0.001) and PACU stay duration (OR = 0.66, 95% CI 0.53–0.82; *p* < 0.001) demonstrated significant associations with pain. PACU stay duration in the regression model reflects a marker of delayed recovery rather than a causal determinant of postoperative pain. The group variable, BMI, and age did not show independent associations with either agitation or pain (Table 5).

In the adjusted multivariate models, BMI and anesthesia duration were included as covariates to control for potential confounding; however, neither variable demonstrated a significant independent association with postoperative agitation or moderate-to-severe pain (all *p* > 0.05), and their inclusion did not alter the direction or statistical significance of the intervention effects (Table 5).

**Figure 3:**
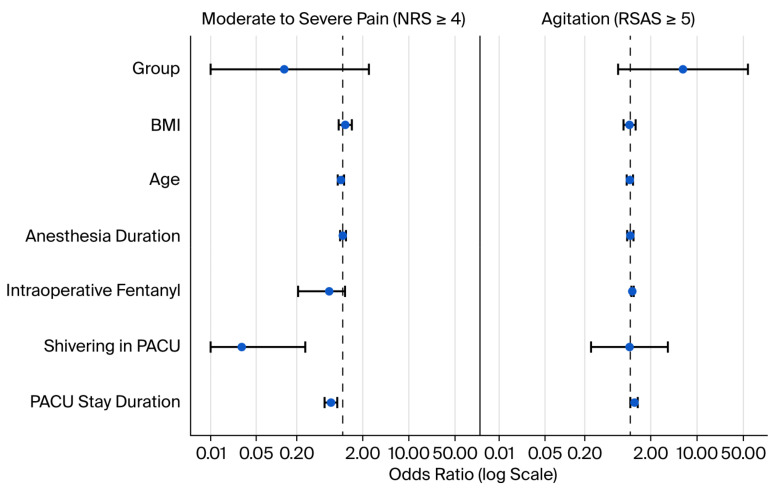
Independent clinical predictors of postoperative agitation and moderate-to-severe pain expressed as odds ratios with 95% confidence intervals.

Collectively, these results demonstrate that active perioperative warming not only maintained intraoperative normothermia but also improved the postoperative recovery quality by reducing shivering, agitation, and pain, while shortening PACU stays (Figure 3).

## 4. Discussion

In this prospective, randomized controlled study, the efficacy of active warming using a resistive carbon fiber heating blanket during the peri-induction period was evaluated in patients undergoing laparoscopic cholecystectomy. Our findings demonstrate that peri-induction active warming improves intraoperative thermal stability and is associated with clinically relevant improvements in postoperative recovery, including reduced pain, shivering, agitation, and shorter PACU stays.

Previous studies consistently demonstrate that active warming is superior to passive insulation in limiting perioperative heat loss [15,16]. Although the absolute temperature differences observed in our study were modest (0.3–0.4 °C), such differences have been shown to be sufficient to meaningfully influence thermoregulatory responses, including shivering and postoperative discomfort, underscoring their clinical relevance. Despite differences in the BMI and anesthesia duration between groups, the superiority of the temperature control in the active warming group remained evident, even in the presence of these potential confounding factors. These findings highlight that applying warming during the early phase of anesthesia—when heat loss is most pronounced—is a critical strategy for preventing hypothermia.

Previous studies investigating warming during the peri-induction period to prevent hypothermia also support our results [11,17]. Horn et al. and Xiao et al., respectively, demonstrated that just 10 and 30 min of prewarming before general anesthesia significantly reduced the incidence of hypothermia [10,18]. However, since pre-warming was not applied in the present study, direct comparison should be interpreted cautiously. Our peri-induction warming protocol represents a practical alternative when pre-warming is not feasible, particularly in fast-turnover surgical settings.

Although anesthesia and surgical durations were relatively short, the first hour of general anesthesia represents the period of greatest redistribution heat loss, accounting for up to 80% of total temperature decline [5]. This study therefore specifically captures the window of maximal thermoregulatory vulnerability, highlighting the importance of initiating warming as early as possible, even during short procedures.

Although a slight temperature decrease was observed in the control group at PACU discharge (TT4), this pattern aligns with the well-known phenomenon of ongoing postoperative redistribution heat loss in patients who do not receive continuous warming support. Mild postoperative hypothermia can remain clinically silent when shivering and hemodynamic status are stable, as seen in our cohort. Importantly, even these small temperature reductions in Group 2 were associated with higher postoperative shivering, pain, and agitation rates, highlighting the clinical relevance of preventing early postoperative heat loss through continuous warming strategies.

In our study, the proportion of patients reporting mild postoperative pain (NRS < 4) was significantly higher in the active warming group than in the passive insulation group (84.6% vs. 50.7%), supporting the role of normothermia in reducing pain intensity. This finding aligns with previous studies demonstrating improved postoperative pain control with perioperative active warming. Consistent with these observations, the incidence of postoperative agitation (RSAS < 5) was also significantly lower in the active warming group (96.9% vs. 80.6%). These results support the concept that maintaining normothermia attenuates sympathetic activation and shivering-related metabolic stress, thereby facilitating smoother emergence and improving early postoperative recovery. These mechanisms provide a plausible physiological explanation for the observed improvements in early recovery quality [1,7,15].

Shivering is the most common clinical manifestation of perioperative hypothermia and can increase oxygen consumption by two- to threefold, thereby raising the cardiac workload. Therefore, maintaining normothermia is considered the most effective strategy for preventing postoperative shivering [19]. In the literature, the superiority of active warming methods for controlling shivering is well established [2,15,16]. In our study, the incidence of shivering was significantly lower in the group that received active warming with an electric blanket. This finding suggests that maintaining thermal stability reduces the burden of oxygen consumption and cardiac stress.

Maintaining normothermia not only preserves physiological stability but also plays a crucial role in improving recovery quality. In our study, the length of stay in the PACU was 24 ± 6 min in the active warming group, compared to 35 ± 8 min in the control group. This finding clearly demonstrates the contribution of effective temperature management to the early recovery process. Similarly, Akelma et al. reported that a prewarming protocol applied in TURP patients significantly reduced PACU stays compared with a control group (31.18 min vs. 45.64 min) [20].

The logistic regression analysis aimed to identify independent clinical markers associated with postoperative agitation and pain. Intraoperative fentanyl dose remained the only independent predictor of agitation, suggesting that higher opioid requirements may reflect increased intraoperative nociception and may paradoxically contribute to agitation during emergence. On the other hand, postoperative shivering and prolonged PACU stay were independently associated with moderate-to-severe pain, indicating that delayed recovery likely serves as a marker of patient discomfort, impaired thermoregulation, and insufficient physiologic stabilization rather than a causal determinant of pain. Importantly, although active warming significantly improved thermoregulatory and recovery parameters, it did not remain a direct independent predictor in multivariate models, supporting the interpretation that warming exerts its benefits indirectly through multiple interrelated pathways such as reduced shivering, lower oxygen demand, and more stable hemodynamics [21]. Taken together, these findings suggest that postoperative pain and agitation may reflect a broader continuum of impaired thermal and physiological homeostasis. Importantly, the lack of an independent association between active warming and outcomes in multivariable models does not negate its clinical benefit, but rather indicates that its effects are likely mediated through interrelated mechanisms such as reduced shivering, lower metabolic stress, and improved recovery dynamics.

Several mechanisms may explain the better analgesia and lower agitation observed in the actively warmed group. Mild perioperative hypothermia activates the sympathetic nervous system and increases circulating catecholamines, thereby enhancing nociceptive signaling and discomfort during emergence. It also triggers shivering, which substantially increases oxygen consumption and contributes to distress. In addition, hypothermia slows hepatic metabolism and enzymatic drug clearance, potentially prolonging residual anesthetic and opioid effects and predisposing patients to delayed recovery and agitation. Moreover, hypothermia increases inflammatory cytokine release, which may heighten nociception and postoperative pain. Conversely, maintaining normothermia preserves metabolic homeostasis, optimizes drug pharmacodynamics, reduces shivering, and facilitates smoother and more stable emergence. These mechanisms collectively help explain the lower postoperative pain and agitation observed in patients who received active warming in our study [21,22,23].

The limitations of our study include its single-center design and the relatively small sample size of ASA I–II patients undergoing laparoscopic cholecystectomy. Because only low-risk ASA I–II patients were included, the generalizability of the findings to higher-risk populations requires further study. The anesthesiologist responsible for managing the warming intervention could not be blinded to group allocation, introducing a potential risk of performance bias, although postoperative outcome assessors remained blinded. Notably, minor baseline differences in BMI and anesthesia duration between groups may have influenced thermoregulation and recovery outcomes, as higher BMI is associated with altered heat distribution, and longer anesthesia duration may increase cumulative heat loss. However, these variables were adjusted for in multivariable analyses and did not independently predict the primary outcomes, suggesting that meaningful confounding is unlikely, although residual confounding cannot be entirely excluded. Tympanic temperature may be influenced by ambient factors and lacks the continuous precision of esophageal or nasopharyngeal monitoring; therefore, our results should be interpreted with this limitation in mind. The absence of depth-of-anesthesia monitoring represents another potential source of bias, as anesthetic depth may influence thermoregulation, opioid requirements, and emergence characteristics. Although standardized anesthetic protocols were applied, unmeasured variability in anesthetic depth may have contributed to postoperative agitation or pain outcomes. Moreover, long-term outcomes and patient satisfaction were not assessed. Nevertheless, the prospective, randomized, and blinded design of this study enhances the reliability of the findings.

## 5. Conclusions

This study demonstrated that active warming using an electric blanket during the peri-induction period effectively maintained core body temperatures during laparoscopic cholecystectomy. Active warming reduced the incidence of postoperative shivering, pain, and agitation and shortened the length of stay in the PACU. Therefore, peri-induction active warming should be considered as a routine component of thermal management in short- and medium-duration laparoscopic surgeries.

## Figures and Tables

**Table 1:** Patient characteristics and intraoperative parameters by study group.

Variable	Group 1 (*n* = 65)	Group 2 (*n* = 67)	Total (*N* = 132)	*p*-Value
Age (years), mean ± SD	45.0 ± 9.8	45.1 ± 9.3	45.1 ± 9.5	0.943
Sex				0.763
Male, *n* (%)	47 (72.3%)	50 (74.6%)	97 (73.5%)	
Female, *n* (%)	18 (27.7%)	17 (25.4%)	35 (26.5%)	
BMI (kg/m^2^), mean ± SD	27.3 ± 3.3	28.8 ± 3.5	28.1 ± 3.5	0.013
Grade of ASA				0.374
ASA I, *n* (%)	20 (30.8%)	16 (23.9%)	36 (27.3%)	
ASA II, *n* (%)	45 (69.2%)	51 (76.1%)	96 (72.7%)	
Rescue fentanyl requirement, n (%)	8 (12.3%)	10 (14.9%)	18 (13.2%)	0.695
Anesthesia time, median [IQR]	74 [70–78]	70 [69–75]	73 [70–77]	0.023
Surgical time, median [IQR]	57 [55–60]	55 [50–60]	56 [52–60]	0.148
Fentanyl (µg), median [IQR]	0 [0–0]	0 [0–0]	0 [0–0]	0.665

Group 1 = active warming group; Group 2 = control group. BMI = body mass index; ASA = American Society of Anesthesiologists Classification; IQR = interquartile range; SD = standard deviation. Anesthesia and surgical times are expressed in minutes. *p* < 0.05 is considered statistically significant.

**Table 2:** Comparison of core temperature at different time points between the active warming and control groups.

Variable	Group 1	Group 2	Repeated Measures F Test		
			*p*-Value	*F*	Partial *η^2^*
TT1 (°C)	36.5 ± 0.55	36.6 ± 0.54	1.00		
TT2 (°C)	36.7 ± 0.53	36.5 ± 0.54	<0.001		
TT3 (°C)	36.6 ± 0.49	36.4 ± 0.54	<0.001		
TT4 (°C)	36.6 ± 0.51	36.2 ± 0.52	<0.001		
Time main effect			<0.001	74.6	0.365
Group main effect			0.039	4.34	0.032
Group × time			<0.001	102.2	0.440

TT1 = baseline (before induction); TT2 = 20 min after induction; TT3 = after extubation; TT4 = upon discharge from PACU. *p* < 0.05 indicates statistical significance.

**Table 3:** Group-wise comparison of sedation (RSAS) and pain (NRS) outcomes.

Outcome	Category	Group 1 (*n* = 65)	Group 2 (*n* = 67)	OR (95% CI)	*p*-Value
RSAS	<5	63 (96.9%)	54 (80.6%)		
	≥5	2 (3.1%)	13 (19.4%)	6.12 (0.64–58.68)	0.116
NRS	<4	55 (84.6%)	34 (50.7%)		
	≥4	10 (15.4%)	33 (49.3%)	0.13 (0.01–2.48)	0.176

NRS = Numerical Rating Scale; RSAS = Riker Sedation–Agitation Scale. RSAS < 5 indicates adequate sedation; NRS < 4 indicates mild or no pain. Odds ratios reflect adjusted estimates derived from multivariable logistic regression analysis.

**Table 4:** Secondary outcomes related to recovery.

Variable	Group 1 (*n* = 65)	Group 2 (*n* = 67)	Total (*N* = 132)	*p*-Value
PACU time, median [IQR]	24 [23–28]	35 [30–40]	30 [24–35]	<0.001
Shivering severity				0.006
No shivering n (%)	52 (80.0%)	36 (53.7%)	88 (66.7%)	
Only piloerection n (%)	10 (15.4%)	22 (32.8%)	32 (24.2%)	
Visible muscular activity n (%)	3 (4.6%)	9 (13.4%)	12 (9.1%)	

PACU = post-anesthesia care unit, IQR = interquartile range. *p* < 0.05 is considered statistically significant.

**Table 5:** Multivariate logistic regression analyses of independent predictors and model fit statistics for postoperative agitation and pain.

Outcome (Group 1 vs. Group 2)	Predictor	OR	(95% CI)	*p*-Value
RSAS ≥ 5	Group	6.12	0.64–58.68	0.116
	BMI	0.95	0.77–1.17	0.619
	Age	0.96	0.89–1.04	0.309
	Anesthesia Duration	0.99	0.88–1.11	0.811
	Intraoperative Fentanyl	1.05	1.02–1.10	0.006
	Shivering in PACU	0.95	0.25–3.65	0.943
	PACU Stay Duration	1.12	0.98–1.28	0.106
NRS ≥ 4	Group	0.13	0.01–2.48	0.176
	BMI	1.10	0.87–1.37	0.422
	Age	0.94	0.84–1.04	0.241
	Anesthesia Duration	0.98	0.90–1.12	0.763
	Intraoperative Fentanyl	0.62	0.21–1.08	0.993
	Shivering in PACU	0.03	0.01–0.27	0.001
	PACU Stay Duration	0.66	0.53–0.82	<0.001

OR = odds ratio; CI = confidence interval; PACU = post-anesthesia care unit; NRS = Numerical Rating Scale; RSAS = Riker Sedation–Agitation Scale. Nagelkerke R^2^ = 0.489 and Hosmer–Lemeshow χ^2^(8) = 6.72, *p* = 0.567 for agitation model; Nagelkerke R^2^ = 0.871 and Hosmer–Lemeshow χ^2^(8) = 4.11, *p* = 0.847 for pain model. *p* < 0.05 was considered statistically significant.

## Data Availability

The data that support the findings of this study are available upon request from the corresponding author.
